# Polysaccharide-based hydrogels for cartilage regeneration

**DOI:** 10.3389/fcell.2024.1444358

**Published:** 2024-10-11

**Authors:** Ning Chen, Sidi Li, Congrui Miao, Qin Zhao, Jinlei Dong, Lianxin Li, Ci Li

**Affiliations:** ^1^ Medical Science and Technology Innovation Center, Shandong First Medical University and Shandong Academy of Medical Sciences, Jinan, Shandong, China; ^2^ College of Chemistry and Chemical Engineering, Yantai University, Yantai, Shandong Province, China; ^3^ Department of Rehabilitation Medicine, Shandong Provincial Hospital Affiliated to Shandong First Medical University, Jinan, Shandong, China; ^4^ Department of Orthopedics, Shandong Provincial Hospital Affiliated to Shandong First Medical University, Jinan, Shandong, China

**Keywords:** cartilage regeneration, polysaccharide, biomaterials, hydrogels, 3D bioprinting

## Abstract

Cartilage defect is one of the common tissue defect clinical diseases and may finally lead to osteoarthritis (OA) which threat patients’ physical and psychological health. Polysaccharide is the main component of extracellular matrix (ECM) in cartilage tissue. In the past decades, polysaccharide-based hydrogels have shown great potential for cartilage regeneration considering unique qualities such as biocompatibility, enhanced cell proliferation, drug delivery, low toxicity, and many others. Structures such as chain length and chain branching make polysaccharides have different physical and chemical properties. In this review, cartilage diseases and current treatment options of polysaccharide-based hydrogels for cartilage defection repair were illustrated. We focus on how components and structures of recently developed materials affect the performance. The challenges and perspectives for polysaccharide-based hydrogels in cartilage repair and regeneration were also discussed in depth.

## Graphical Abstract

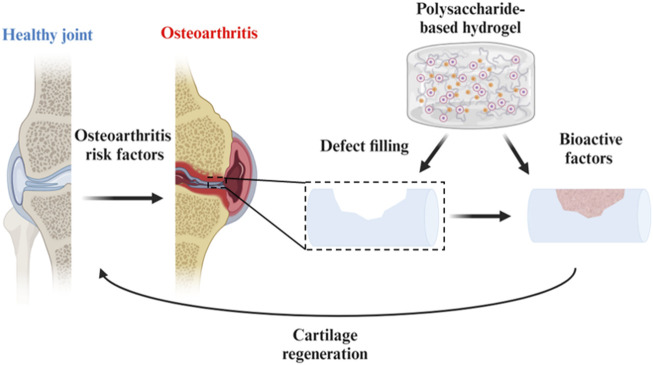

## 1 Introduction

Osteoarthritis (OA) is one of the most common chronic joint diseases today. Patients with OA often suffer from joint pain, joint stiffness, and disability of joints (Murphy et al., 2020). Unlike bone tissue, cartilage is a hierarchically organized, porous, viscoelastic tissue with a dense extracellular matrix (ECM) but has a limited intrinsic regenerative ability due to the low cell density, lack of vasculature, nervous and lymphatic systems (Abdulghani and Morouço, 2019). As for mature chondrocytes, the matrix is produced at a slow rate. In clinics, typical treatments are surgical operations including autologous chondrocyte implantation (ACI), matrix-associated autologous chondrocyte implantation (MACI) and microfracture (Tsanaktsidou et al., 2022), which are characterized by low patient compliance. Therefore, it is urgent to explore useful therapeutic methods that can intervene early in the degeneration and extend the service life of articular cartilage (Wongin et al., 2021).

Biomaterials, a kind of biological material without zoonotic transmission in contact with tissues or biological fluids, have attracted considerable attention from human beings in the last few decades (Su et al., 2021). Among the numerous biomaterials, hydrogels are widely applied in tissue engineering regeneration. The three-dimensional network structure gives hydrogels the characteristic of containing huge amounts of water. Besides, the hydrogels can present different physical and biochemical characteristics because of the diversity of materials, flexible designability, and controllable crosslinking density. Many natural and/or synthetic polymers and numerous kinds of crosslinking methods have been used to design hydrogels for different biomedical applications, such as drug delivery, tissue adhesives, and space filling (Guo et al., 2022; Zhang et al., 2023). To give full play to the distinctive biocompatible and non-toxic nature of hydrogels, the raw materials for making hydrogels have received extensive attention. The combination of hydrogels with cells, growth factors, or artificial substrates has great potential to improve cartilage regeneration (Yuan et al., 2021).

Polysaccharide [the general formula is Cx (H_2_O)n] is a common biomacromolecule substance in nature. For most living organisms, polysaccharides are the most important substances for maintaining vital activities. Therefore, polysaccharide-based hydrogels have been widely applied in biomedicine and nanobiotechnology fields due to their definite advantages over synthetic polymers such as biocompatibility, biodegradability, nontoxicity, and ease of modification (Dhawan and Cui, 2022). Besides, the components, the length of molecular chains and the difference of chain branching make polysaccharides have different physical and chemical properties (Raina et al., 2022). Thanks to these unique properties, polysaccharide-based hydrogels have been widely used in cartilage repair therapy. Representative polysaccharides include hyaluronic acid, alginate, chitosan, and chondroitin sulfate.

In this review, we focus on summarizing the latest progress in cartilage defects and the recent advances of polysaccharide-based biomaterials in cartilage regeneration. Firstly, the biological features of OA and cartilage regeneration are discussed. And then, the present therapeutic strategies for cartilage regeneration are shown in the review. Finally, the developments of designing various polysaccharide-based hydrogels for cartilage defection therapy are revealed.

## 2 The biological characteristics of OA and cartilage regeneration

**FIGURE 1:**
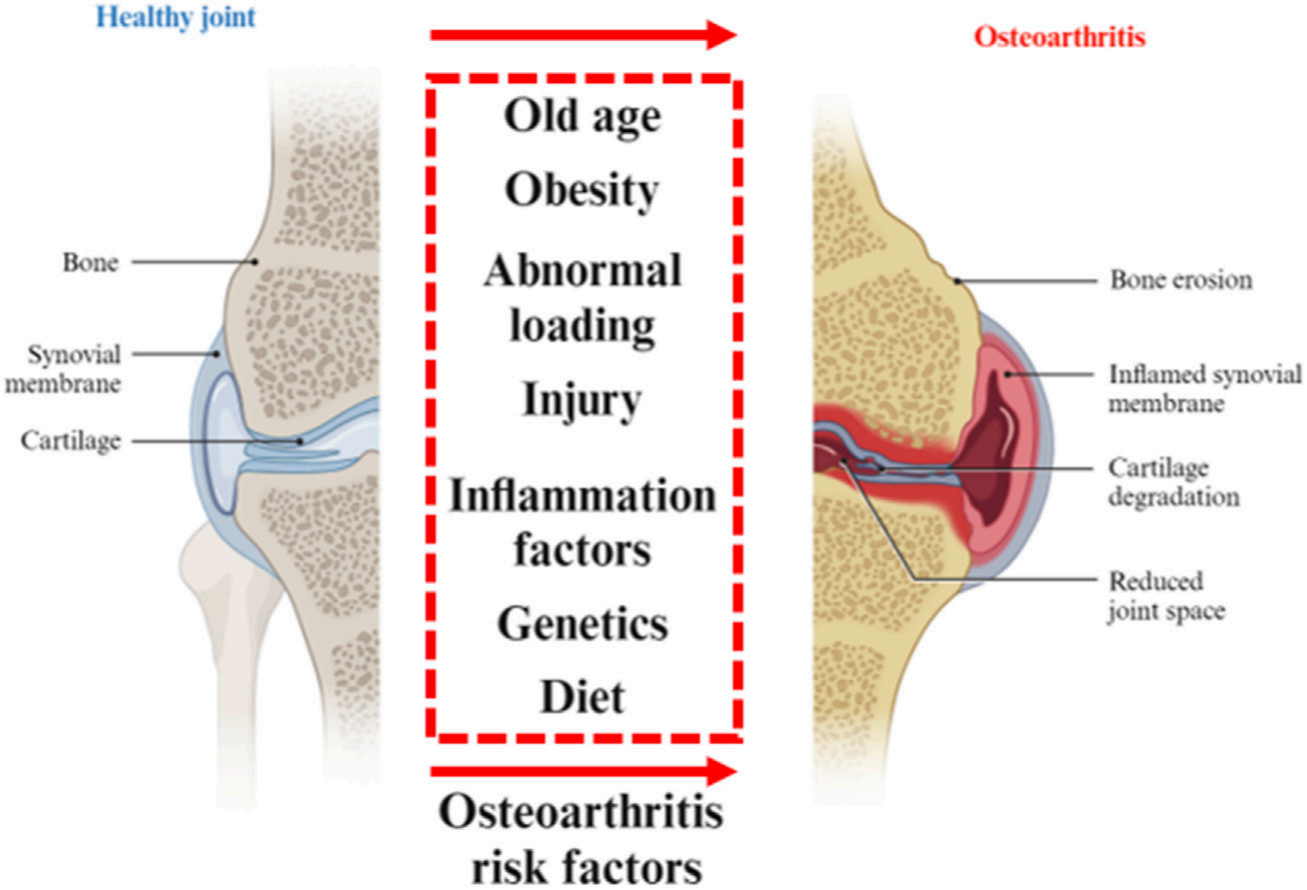
Osteoarthritis risk factors. Various risk factors for healthy joints to degenerate into osteoarthritis. Partially designed with BioRender.

Except for “wear and tear” of the joint, the inflammatory and metabolic factors are thought to be important causes of joint stiffness, swelling and loss of mobility. The progression of OA affects the entire joint tissue. OA develops from the breakdown of the cartilage matrix. After the degenerative ECM becomes fibrillation and erosion, the collagen fragments activate the inflammation of synovitis that induces further cartilage degradation. Besides, joint ligaments and subchondral bone also participate in the inflammatory response. The most notable for the pathogenesis of OA is the cartilage injury. In physical factors, continuous load-bearing and friction will thin the cartilage. The aging procession including DNA damage, mitochondrial dysfunction, and autophagy limits the capacity of self-renewal of chondrocytes. The production of metalloproteases, synovial angiogenesis, and inflammatory cytokines, which accelerate cartilage destruction (Martel-Pelletier et al., 2016; Xia et al., 2014) (Figure 1).

Chondrocytes are specific cells in cartilage tissue (3%–5% of articular cartilage tissue) that are present in high-density extracellular matrix (ECM) (Heinegård and Saxne, 2011). Mesenchymal stem cells (MSCs) are involved in the formation and differentiation of chondrocytes. In mature cartilage tissues, stem cells generally gather in the deep layer of perichondrium and have the potential to differentiate into chondrocytes again (Xiang et al., 2022). In addition to the chondrocytes and MSCs, the ECM also are the indispensable composition of the organization of cartilage tissue. The ECM provides a suitable microenvironment for the proliferation, differentiation and maintenance of physiological activities of various cells in cartilage tissue (Benders et al., 2013). The ECM of cartilage tissue is a unique tissue-specific 3D environment composed of multiple types of collagens and proteoglycans. The ECM of cartilage tissue is a unique tissue-specific 3D environment composed of multiple types of collagen and glycans. Fibrous collagen forms the network structure of the ECM and contains a variety of bioactive factors, such as growth factors, integrins and functional peptides. ECM provides load bearing, joint lubrication and joint connection for cartilage tissue. And in addition to fibrous collagen, the network structure of ECM also contains glycoglycans, such as aggregin, keratin sulfate glycosaminoglycans, hyaluronic acid, chondroitin fate glycosaminoglycans and glycoprotein lubricant (PRG4). These unique proteins and bioactive factors can respond to external dynamic events and form intricate interactions between cells (Bhattacharjee et al., 2015) Therefore, resorting to cartilage tissue elements is a promising approach to promote cartilage injury recovery.

## 3 Therapeutic strategies for promoting cartilage regeneration

### 3.1 The types of bioactive factors

After cartilage tissue injury, it is difficult to achieve satisfactory healing only by the repair of injured marginal tissue, and additional biochemical and mechanical signals are needed to stimulate cells into the regeneration process (Liu Y. et al., 2022). In this review, the several bioactive factors or drugs that assist cartilage regeneration, and the recent studies related to enhancing cartilage regeneration tissue were summarized (Table 1).

**TABLE 1:** The bioactive factors or drugs to assist cartilage regeneration.

Factors	*In vitro* (cell type)/*In vivo* (animal type) testing	Mechanism of action results featured	Ref.
Drugs Chondroitin sulfate	*In vitro* (rat adipose-derived stem cells)	Chondroitin sulfate maintains the structural integrity of cartilage and restores arthritic joint function because of its antiinflammatory activity stem cell niche maintenance, and regulating enzyme activity. The mechanical property and biochemical properties presented tunable properties which offer attractive flexibility for cartilage regeneration	Li et al. (2021a)
Metformin and strontium	*In vitro* (Chondrocytes, from an old patient with n late-staged OA)/*In vivo* (Sprague-Dawley rats)	Metformin inhibits senescence and strontium is an anti-inflammatory material. Accelerated cartilage repairment, and inhibited chondrocyte senescence were significant	(Xu et al., 2021a)
Corticosteroids	*In vitro* (Chondrocytes, Patient with OA)/*In vivo* (Sprague-Dawley rats, Patient with OA)	Corticosteroids block phospholipase A2, thereby inhibiting the arachidonic acid cascade and the generation of kinins, prostaglandins, and other inflammatory mediators. And low dose corticosteroids exert effects on down-regulating the gene expression level of MMPs	(Bannuru et al., 2019), (Kou et al., 2019)
Extracellular substance Engineered exosomes	*In vitro* (Synovial fluidderived mesenchymal stem cells)/*In vivo* (Sprague-Dawley rats)	Targeted delivery of Kartogenin to synovial fluid-derived mesenchymal stem cells and leads to even dispersion of KGN in the cytosol, increases its effective concentration in 8 the cell. Engineered exosomes promote the chondrogenesis of synovial fluid-derived mesenchymal stem cells. Co-administration of SFMSCs with E7-Exo/KGN shows more pronounced therapeutic effects in a rat OA model than KGN alone or KGN delivered by exosomes without E7	(Xu et al., 2021b)
Platelet-rich plasma (PRP)	*In vitro* (Leukocyte and platelet-rich plasma, adipose mesenchymal stem cells)/*In vivo* (Rabbit)	PRP delivers a myriad of growth factors to the injection site, most notably TGF-β, PDGF, FGF, VEGF, and IGF. The delivery of these growth factors to the joint can have a positive anti-inflammatory benefit. PRP also promote the differentiation of adipose mesenchymal stem cells into mature cartilage	(Beigi et al., 2018), (Hu et al., 2020)
Mesenchymal stem cell derived extracellular matrix (MSC-ECM)	*In vitro* (Human knee articular chondrocytes)/*In vivo* [Female Severe Combined Immunodeficiency (CB17/IcrPrkdcscid/IcrIcoCrl SCID) mice]	As a culture substrate for chondrocyte expansion *in vitro*, as well as a scaffold for chondrocyte-based cartilage repair, promote MSC adhesion, proliferation, chondrogenic potential, as well as cartilage matrix deposition compared to MSCs in pellet culture. Prominent cartilage formation was observed in the implanted Cell/ECM constructs 14 days postimplantation, with higher sGAG deposition compared to controls consisting of chondrocyte cell sheets	(Yang et al., 2018a)
Cells skeletal stem cells	*In vitro* (Skeletal stem cells)/*In vivo* (Mouse)	Local acute injury activated skeletal stem cells to enhance chondrogenic potential The resident skeletal stem cells can be induced to generate cartilage	(Murphy et al., 2020)
Allogeneicinduced pluripotent stem cell (iPSC)	*In vitro* (iPSC)/*In vivo* (Rat)	iPSCs have unique pluripotency and selfrenewal properties The self-renewal ability of iPS cells enables an unlimited supply of allogeneic iPSC-derived cartilage, solving the problems of allogeneic cartilage, such as the scarcity of donors and variations in cartilage quality among donors	(Abe and Tsumaki, 2023)
Macrophages	*In vitro* (Macrophages)/*In vivo* (Rat)	M_2_ macrophage polarization and inhibit the inflammatory reaction. M_2_ macrophages maintain the balance of the inflammatory microenvironment and promote cartilage regeneration	(Xiao et al., 2023)
Growth factors miRNA	*In vitro* (Chondrocytes)/*In vivo* (Rat)	The miR-29b-5p created a regenerative microenvironment to mitigate chondrocyte senescence. miR-29b-5p recruited of synovial stem cells and promote synovial stem cells differentiation into chondrocytes	(Zhu et al., 2022)
TGF-β and Sox9	*In vitro* (Human normal and OA chondrocytes and cartilage explants)	TGF-β and Sox9 promote the mitogenic and proanabolic properties. TGF-β and Sox9 enhanced the levels of cell proliferation both in human normal and OA chondrocytes and cartilage explants	(Song and Park, 2020), (Tao et al., 2016)
Kartogenin	*In vitro* (Cartilagederived-stem/progenitor cells)/*In vivo* (Rat)mice OA model with destabilization of medial meniscus	Kartogenin can decrease the levels of nitric oxide and glycosaminoglycans (GAGs) Kartogenin can maintain chondrocyte phenotypes but also protects the cartilage matrix from degradation	(Zhang et al., 2019)

### 3.2 The current methods for controllable release

Methods to prolong the usage time of articular cartilage include oral or intra- articular injection of drugs, but they may face significant obstacles in terms of persistence of effect and medical compliance. However, remarkable achievements will be made in cartilage injury repair and joint function recovery, if chondrocytes and MSCs can be scientifically and effectively induced to regenerate and differentiate. With the development of tissue engineering research, biomaterials have become an important application point to support cartilage regeneration due to their excellent biocompatibility, degradability, and controllable mechanical strength (Makris et al., 2015; Mokhtarinia and Masaeli, 2023; Zhou et al., 2022).

## 4 Polysaccharide-based hydrogels

### 4.1 Design of polysaccharide-based materials

To mimic the structure of cartilage tissue and physicochemical properties of the ECM as well as possible, several parameters of the hydrogels should be considered: 1) good biocompatibility; 2) adequate mechanical properties; 3) various chemical modifiability; 4) promoting cell adhesion; 5) biological functionality, integration with defect site; 6) sufficient porosity and pore interconnectivity to allow cell-mediated matrix deposition and angiogenesis (Fu et al., 2021; Ghanbari et al., 2021a; Hiew et al., 2018). Polysaccharide based hydrogels are ideal candidates for constructing bionic extracellular matrix (ECM) because of their bionic network, high water content, biocompatibility, and biodegradability. Nowadays, the main polysaccharide materials, which were used to construct hydrogel scaffolds, 149 include hyaluronic acid (HA), alginate, chitosan, and chondroitin sulfate Polysaccharide-based hydrogels can be loaded with bioactive molecules, growth factors, exosomes, or cells, to further enhance cartilage regeneration. These bioactive agents could promote chondrogenesis, inhibit inflammation, or stimulate angiogenesis, depending on the specific requirements of the cartilage repair process. In the following section, the features, limitations, and modification strategies of each kind of polysaccharide were introduced.

#### 4.1.1 Hyaluronic acid

HA, widely distributed in the soft tissues, is an anionic polysaccharide playing crucial roles in cell transduction and matrix formation. HA possesses special biological actives for fabricating hydrogel scaffolds interaction with cells as follows. 1) Joint lubrication: the combination of HA derivatives with cartilage tissue precisely mimics the lubrication and viscoelastic functions of lubricating proteins on the surface of cartilage, playing a protective role in joints (Singh et al., 2014). 2) Anti-inflammatory effect: It was found that the scaffold surface modified by HA derivatives could inhibit the aggregation of inflammatory cells at the lesion site, and reduce the release and activity of pro- inflammatory factors and mediators by suppressing the interaction between inflammatory key receptor and their ligands on the cell membrane (Knopf-Marques et al., 2016). 3) Cartilage regeneration: HA also could interact with MSCs via cell surface receptors including CD44, ICAM-1 and RHAMM, and is benefit for accumulating substantia cartilaginous matrix (Chung and Burdick, 2009; Nettles et al., 2004). HA is involved in cell proliferation, morphogenesis, inflammation, and wound repair and is an ideal choice for manufacture of chondrocyte carrier materials in cartilage regeneration.

**FIGURE 2:**
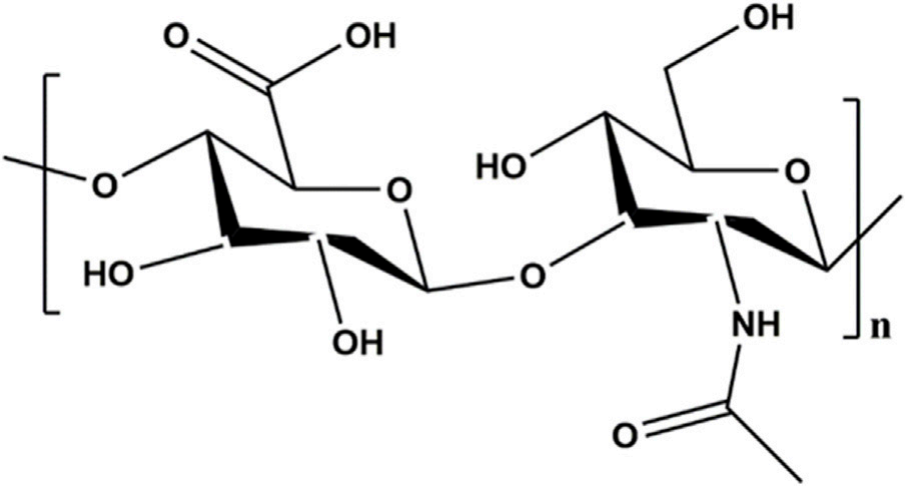
The structure of HA.

The linear structure of HA is shown in Figure 2. HA possesses a large number of hydrophilic active functional groups, such as free hydroxyl, carboxyl and acetylamino groups, which are readily modified with sulfhydryl group and allyl group (Campoccia et al., 1998; Wang M. et al., 2022). These characteristics make HA a desirable candidate for designing carrier materials loaded with cells, drugs or other active factors. Typically, thiol-modified hyaluronic acid, collectively named HA-SH, was prepared by carbodiimide-mediated hydrazide chemistry and then crosslinked with vinyl sulfonated triblock copolymer to form hydrogels by Michael addition (Censi et al., 2020). Given the diverse modifiability of HA, increasing studies adopted HA and HA derivatives as the matrix materials in the upper or lower layers to synthesize biphasic scaffolds (Liu et al., 2020; Wang et al., 2021). For instance, HA hydrogels of different physicochemical and biological properties can be infused into the 3D scaffold to form a bilayer scaffold, realizing the repair of osteochondral defects with different structures (Chen et al., 2022). The dynamic HA layer exhibited favorable stress relaxation due to the cross-linking kinetics of HA hydrazone for cartilage regeneration and the porous HA layer provided mechanical support, maintained long-term mechanical stability and promoted cell adhesion and proliferation for subchondral bone repair.

Considering the unique rigid cylindrical spiral shape of HA spatial structure, HA hydrogels have special physical and chemical properties, such as good hydrophilicity, strong water absorption and retention, high biocompatibility, biodegradability, viscoelasticity, printing flexible (Yin et al., 2018; Zhu et al., 2018). HA hydrogels with high viscosity play the essential role in protecting articular cartilage surface from shear stress. However, the chief shortcomings of HA alone to construct hydrogels are the poor mechanical properties and fast biodegradability, which finally lead to the unsustainable cartilage tissue repair. To solve these problems, many efforts have been devoted to improve mechanical strength and viscoelasticity, and resistance to rapid degradation nowadays.

Methacrylated hyaluronic acid (HAMA) is a typical example of a modified HA to present increased rigidity and higher resistance to degradation (Burdick et al., 2005; Poldervaart et al., 2017). With a photo initiator, different concentrations and molecular weights of HAMA can be crosslinked and form hydrogels under UV irradiation. The results showed that the compressive moduli of 50 kDa 20 wt% HAMA was about kPa, which was much higher than that of 50 kDa 2 wt% HAMA (∼12 kPa). Besides, the complete degradation time of 10 wt% of MAHA hydrogel with 50% uronic acid was almost 20 days, but that of 5wt% of MAHA hydrogel with 40% uronic acid was less than 2 days.

**FIGURE 3:**
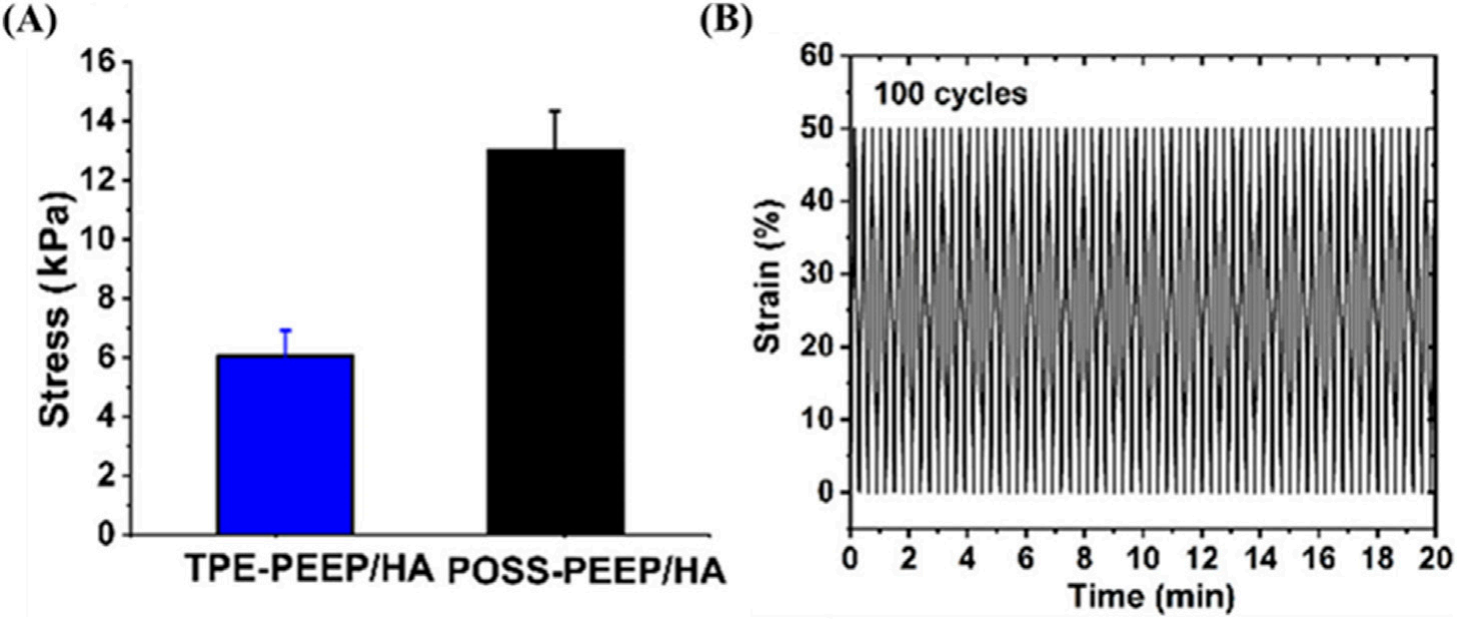
The mechanical strength of POSS-PEEP/HA. **(A)** Compressive modulus of POSS-PEEP/HA and TPE-PEEP/HA hydrogels. **(B)** Recovery cyclic tests from 50% to 0%. Copyright from ACS publications. (Reprinted with permission from Cui et al., 2023. Copyright 2023 American Chemical Society).

Introducing nanoparticles or reinforcing materials could enhance the mechanical properties of HA based hydrogels. It has been reported that pluronic F127 diacrylate (F127DA) nano-micelles with sizes of 10–20 nm serve as the macro-crosslinkers were added to improve the mechanical performance of hyaluronate hydrogels under photo-polymerization (Ren et al., 2019). Besides, graphene (GR) also could present strength mechanical strength of scaffolds. Compared with TPE-PEEP/HA hydrogel, POSS-PEEP/HA presented higher sheer modulus, compressive modulus, and tensile modulus. Polyhedral oligomeric silsesquioxane (POSS), a kind of rigid inorganic nanocage, also allowed for improving mechanical strength. For another example, an injectable, biodegradable and mechanically reinforced POSS-PEEP/HA hybrid hydrogel scaffold based on polysaccharide HA was synthesized for cartilage regeneration (Cui et al., 2023). Briefly, the hydrogel was synthesized by Michael addition reaction using POSS-8PEEP-AC as core and HA-SH as crosslinker. Compared with pure TPE-8PEEP/HA hydrogel, POSS- 8PEEP/HA hydrogel presented favorable compressibility and fatigue resistance in the cyclic compression experiment and repeated bending properties (Figure 3). Those improved mechanical properties can be ascribed to that the component of POSS could restrict the movement of molecular chains, whose structure is rigid 3D inorganic silica nanocage. And the high survival rate of hMSCs encapsulated in POSS-8PEEP/HA hydrogel shows that POSS-8PEEP/HA hydrogel system has good biocompatibility and provides a 3D platform for cartilage cell adhesion and proliferation. Finally, hMSCs- laden hydrogels were administrated to cartilage defect-induced rats model and effectively promoted cartilage regeneration *in vivo*. Besides, large amounts of studies improve the mechanical properties by constructing double network (DN) hydrogel. DN network consisting of two asymmetric networks such as chemical bond and reversible noncovalent bond. For instance, Cai et al. (2022) synthesized the DN hydrogel based on HA and gellan gum (GG). The first chemical crosslinking network was formed by HA-furan derivatives and 4-arm- maleimido-poly (ethylene glycol) and then GG crosslinked by Ca^2+^ was used as the second ionic crosslinking network to improve the compressive strength and fatigue resistance.

#### 4.1.2 Alginate

Alginate is a natural linear polysaccharide extracted from brown algae, bacteria or kelp, consisting of repeating units of β-1,4-linked D-mannuronic acid (M) and L guluronic acid (G) linked by 1,4-glycosidic bond. Alginate is a copolymer was consisted of a certain proportion of GM, MM, and GG. The number and structure of M and G is different depending on the sources, which would influence the physical and chemical properties. Typically, M segment has better biocompatibility, while G could provide higher rigidity. Therefore, the alginate gel rich in G units has fewer elastic chain segments and higher hardness. Increasing the content of G segment can improve mechanical properties and compressive modulus, making it suitable for repairing cartilage tissue (Liu W. et al., 2022; Zhang and Zhao, 2020).

One of the key advantages of alginate hydrogels is excellent biocompatibility, especially for the hydrogel rich in G segment, which allows for cell encapsulation and proliferation within the hydrogel matrix. Chondrocytes, the specialized cells found in cartilage, can be encapsulated in the alginate hydrogel and cultured to promote cartilage regeneration (Rastogi and Kandasubramanian, 2019). The hydrogel provides a supportive environment for cell growth, nutrient exchange, and extracellular matrix production. Furthermore, alginate hydrogels have the advantage of being injectable, allowing for minimally invasive delivery into cartilage defects or joint spaces. This injectability enables precise placement and full coverage of the damaged tissue, facilitating the regeneration process. Besides, the alginate hydrogels could also be used as 3D biological ink to compensate for cartilage defects (Liu W. et al., 2022).

However, the disadvantage of alginate hydrogels also limits its application in clinic. On the one hand, inherent adhesive performance that may be necessary for long-term integration with surrounding tissue are weak. On other hand, the sodium alginate scaffold cannot provide sufficient and effective mechanical support in pressure environments.

The porous hybrid hydrogels were synthesized by both ionic crosslink network from calcium-alginate non covalent bonding and covalent crosslink network from methacrylate radical polymerization (Stagnaro et al., 2018). The results indicated that the mechanical properties of hybrid EGDMA/HEMA hydrogel (G modulus approached 250 kPa) were much higher than that of alginate hydrogel alone (about 35 kPa). Besides, the characteristic of the double network hydrogels is easy to operate when the first network formed, such as injectability and printability, and the mechanical strength and structural stability of the hydrogel enhanced when the second crosslinking network formed (Asohan et al., 2022). A strategy named confined-chain-aggregation (CCA) was adopted to fabricate ultrastrong and tough hydrogels based on the double network (Xu C. Z. et al., 2021). After the first crosslinked network of polyacrylamide (PAAM) is formed, both the alginate chains and PAAM chains were restricted and the micron-scale aggregates were generated by solvent- induced phase separation. Before ionic crosslinking of alginate. After absorbing water, large-scale cross-linked regions were formed by hydrogen bonding and metal coordination. The results indicated the mechanical strength and toughness of PAAM/Alg hydrogel was five times higher than that of traditional methods, because of the combination of CCA and cationic crosslinking.

**FIGURE 4:**
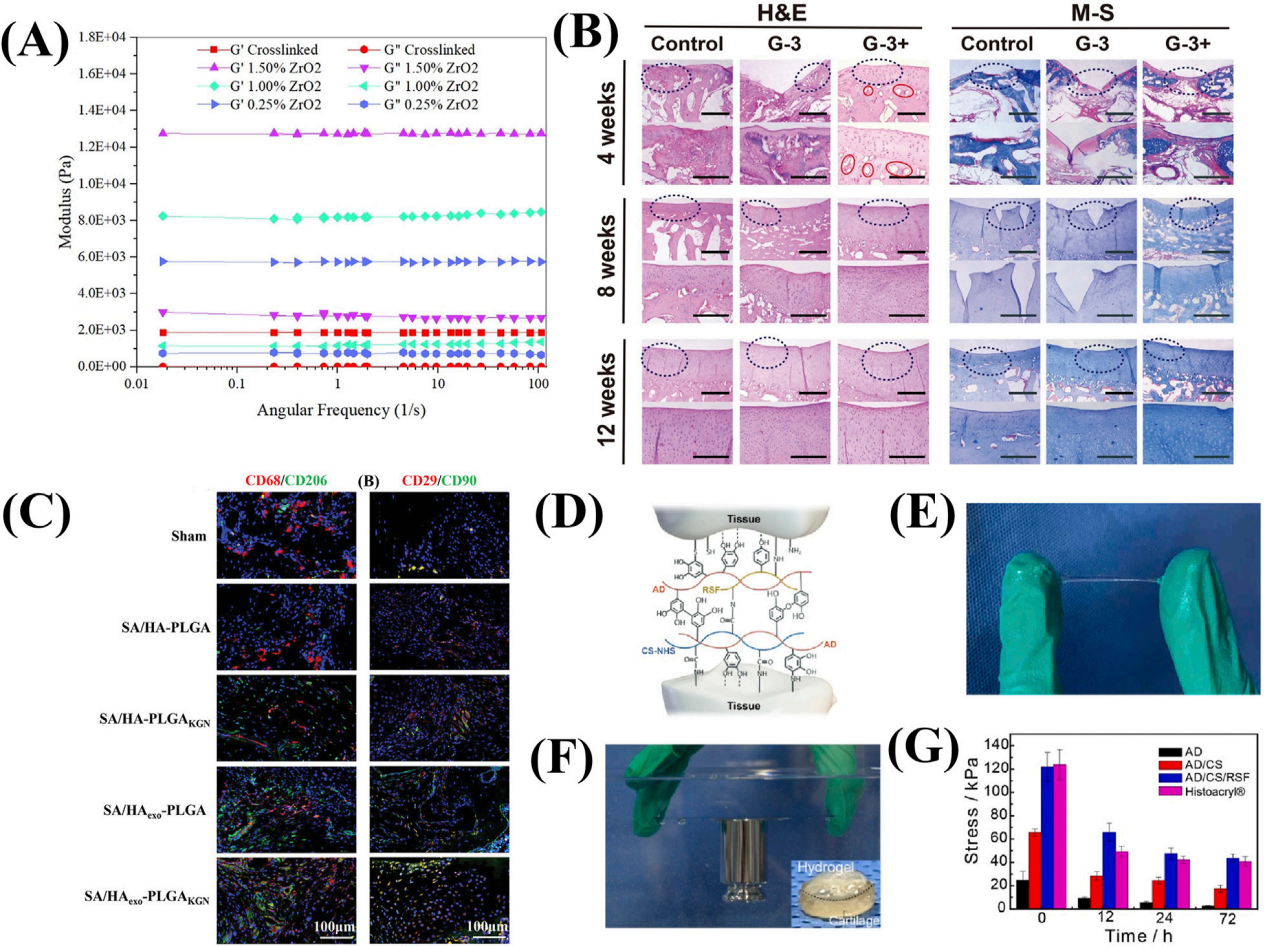
The mechanical strength and the cartilage repair performance of alginate based hydrogels. **(A)** Modulus of hydrogels with different amounts of ZrO2. (Reprinted from Ghanbari et al., 2021b, with permission from Elsevier). **(B)** H&E and M-S histochemical staining images of the regeneration tissues. (Reprinted from Xing et al., 2021, with permission from Elsevier). **(C)** Representative immunofluorescence staining images of CD68 and CD29 (red), CD206 and CD90. (green), and DAPI (blue) in tissue samples collected from the wounds treated with different hydrogels at week 4 after surgery. (Reprinted from Ma et al., 2022, with permission from Wiley). **(D)** Diagram of interaction between tissue residues and the functional groups of AD/CE/RSF hydrogel. (Reprinted from Zhang F. X. et al., 2021, with permission from Elsevier). **(E)** Images of the stretched hydrogel between the fingers. **(F)** The hydrogel on glass in underwater conditions. (Reprinted from Zhang F. X. et al., 2021, with permission from Elsevier). **(G)** Adhesion strength of hydrogels with different time. (Reprinted from Zhang F. X. et al., 2021, with permission from Elsevier).

Introducing reinforcing agents is also a major method to enhance mechanical properties. The injectable and biodegradable hydrogels based on oxidized alginate/gelatin and nitrogen doped carbon dots (NCDs) has been designed as the reinforcing agent (Ghanbari et al., 2021a). Besides, the study also investigated that oxidized alginate based nanocomposite hydrogels containing high ZrO2 NP content (1.5%) as reinforcements had higher mechanical properties than those without NP (Figure 4A) (Ghanbari et al., 2021b).

Osteochondral injury is the focal damaged area of both cartilage and an underlying bone fragment. A single performance scaffolds hardly meet the needs of two types of defect areas simultaneously. The bilayer scaffolds could mimic the structure of cartilage and osteochondral bone. In detail, gum and sodium alginate serve as the cartilage layer, while gum and hydroxyapatite serve as the subchondral layer (Xing et al., 2021). This integrated construct is compatibility and can be loaded together with MSCs, which are associated with the expression of different functional proteins involved in cartilage and bone formation. In the rabbit osteochondral defect model, the hydrogel was used as calcium reservoir to promote angiogenesis and critical defect repair within 8 weeks (Figure 4B).

In addition, introducing adhesive polymers (such as hyaluronic acid and dopamine) can also compensate for the insufficient cell adhesion ability of alginate (Figure 4C) (Ma et al., 2022). Zhang F. X. et al. (2021) fabricated the injectable mussel-inspired highly adhesive hydrogel (AS/CS/RSF) with the crosslinking network of alginate-dopamine, chondroitin sulfate, and regenerated silk fibroin. Due to the nature of the chemical functional residues of AS/CS/RSF hydrogels, such as dopamine, CS-NHS, RSF tyrosine, amino groups and carboxyl groups, there are multiple noncovalent interactions and covalent bonds (Figure 4D). The sulfhydryl and amine groups existing on the surface of these extracellular matrix (ECM) react with NHS, and catecholamine and phenolic hydroxyl groups on the hydrogel to form a covalent cross-linking network, which helps the adhesion of hydrogels. AD/CS/RSF hydrogel provides 120 kPa relative lap shear strength and higher ability to maintain adhesive strength than the commercial tissue adhesive (Figures 4E–G).

#### 4.1.3 Chitosan

**FIGURE 5:**
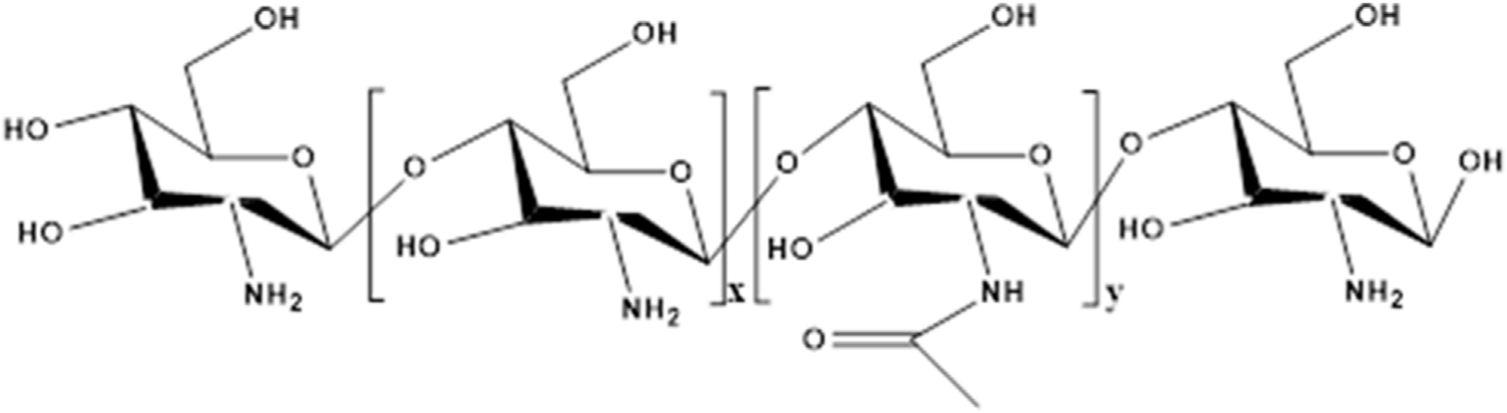
The structure of chitosan. x represents the number of repeating units of glucosamine, y represents the number of repeating units of acetylglucosamine.

Chitosan is a product of N-deacetylation of chitin, which widely exisits in the shell of crustaceans, like shrimp, exoskeletons of mollusk’s and in the cell walls of fungi (Zhou et al., 2008). It is a natural polysaccharide and composed of N-acetylglucosamine and the linear chain of D-glucosamine units through β-glycosidic bond connected together (Figure 5). The amino group in the molecular structure of chitosan is more reactive than the acetylamino group in chitin, which makes the polysaccharide have excellent biological functions and can be chemically modified, including acylation, alkylation, 315 carboxymethylation and carboxyalkylation (Bhattarai et al., 2010). Besides, positively charged chitosan can be bind with negatively charged glycosaminoglycans secreted by chondrocytes to form ion complexes that fix them on the scaffold (Man et al., 2016). Therefore, chitosan-based hydrogel has numerous physiological functions, making it attractive for cartilage repair and regeneration such as biodegradability, biocompatibility, non-toxic, natural broad- spectrum antimicrobial activity, high bio-adhesive, and anti-inflammatory. Composite chitosan-based biomaterials have been demonstrated to stimulate *in vivo* regeneration and the repair of cartilage (Kapat et al., 2019). Therefore, chitosan is considered as a functional biomaterial with great application prospects.

Although chitosan has shown the ability to promote chondrocyte proliferation, maintain normal chondrocyte phenotype, and induce adipose derived stem cells (hASCs) to differentiate into chondrocytes *in vivo* and *in vitro* (Asgarpour et al., 2021), the conventional chitosan-based hydrogels generated from an acidic solvent yield exhibit weak mechanical properties and low stability (Fang et al., 2022). Introducing reinforcing material is an option to improve its properties for wider applications. For instance, CNCs as nanofillers were introduced into CS/pectin hydrogel to enhance the mechanical properties. With the increase of the content of CNCs, the network structure of the composite hydrogels was more compact and the equilibrium swelling ratio decreased. The storage modulus (G ′) of hydrogels with high content of AD CNCs (0.5 wt% CNCs) presented 2.5–3 times higher than that of the hydrogel without CNCs (Ghorbani et al., 2020).

In addition, chitosan lacks integration with the host bone, which becomes a major challenge in repairing subchondral defects. Recently, chitosan-based fiber scaffolds are obtained by electrospinning as a scaffold for chondrocyte growth. Compared with thin films, higher ratio of living chondrocyte on fibers within the first 8 h after inoculation indicated that the morphology of the matrix is a key impact on the interaction between cells and scaffolds (Garcia et al., 2021).

#### 4.1.4 Chondroitin sulfate

**FIGURE 6:**
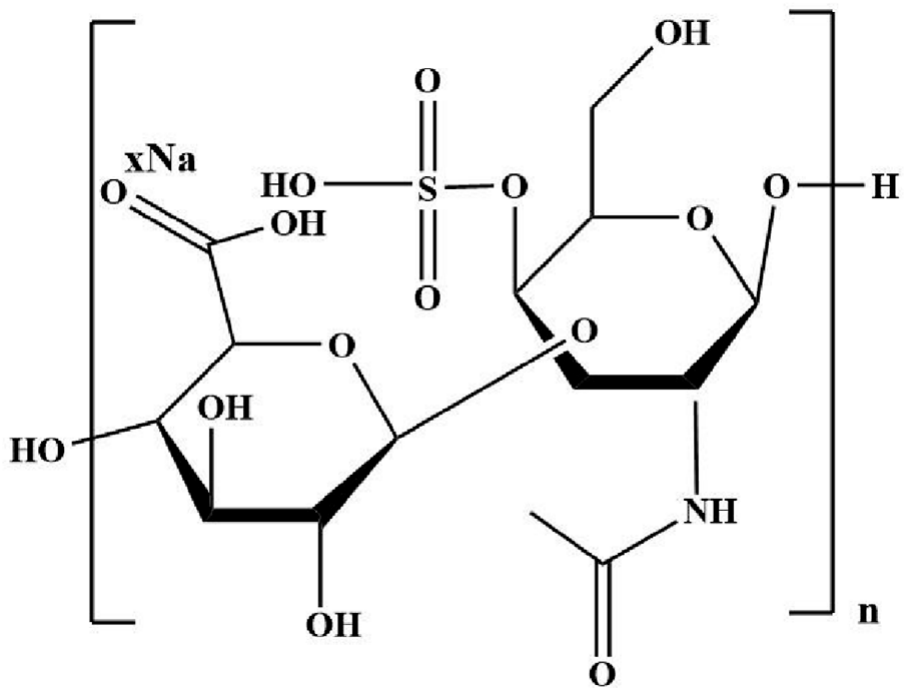
The structure of chondroitin sulfate.

Chondroitin sulfate, composed of D-glucuronic acid and N-acetyl-D-galactamine units (Figure 6), is the glycosaminoglycan extracted from the extracellular matrix of animal tissues such as connective tissue, bones, ligaments, tendons, and cartilage. Chondroitin sulfate based hydrogels are widely used as tissue engineering scaffolds because of their good biocompatibility, biodegradability and similar structure to natural cartilage ECM (Wang and Yang, 2017). Chondroitin sulfate contains a large number of hydroxyl, carboxyl, and sulfonic groups, which endows them with excellent hydrophilicity and chemically modify (Zhang Q. et al., 2021). Besides, chondroitin sulfate, as a natural polymer, has anti-inflammatory activity and can effectively promote the synthesis of proteoglycans and hyaluronic acid, reduce the metabolic activity of chondrocytes, enhance the specific gene expression of chondrocytes, and inhibit the synthesis of proteolytic enzymes (Gao et al., 2018) It is very beneficial for reducing symptoms of osteoarthritis. Chondroitin sulfate has the ability to enhance the secretion of proteoglycans and collage. This endows it with the potential to treat cartilage tissue defects in clinic. The interpenetrating network hydrogel with chondroitin sulfate, gelatin and HA as the main components was prepared by click chemical method (Yu et al., 2013). Hyaluronic acid and gelatin were respectively modified using furan methylamine and furan formic acid, and then were mixed with maleimide polyethylene glycol (MAL-PEG-MAL) to generate the first network by Diels-Alder reaction. Finally, the secondary network was formed after adding chondroitin sulfate. Because neither additional organic crosslinking agent nor catalyst was required, the hydrogel is non-toxic and harmless. It is an effective method to prepare cartilage tissue engineering scaffolds.

### 4.2 Technologies for synthesizing hydrogels

There is an increasing demand for fabricating new technologies and good outcomes to satisfy irregularly shaped cartilage defects. The methods are listed as follows in Table 2.

**TABLE 2:** The methods of synthesizing hydrogel scaffolds.

Technology	Operational approach	Advantages	Ref.
Injectable technology	Bulk mixing	Easy to operate Minimally invasive Complete filling of the defect site	(Chen et al., 2022)
3D bioprinting technology	3D bioprinter printing	Precise control of cell distribution and growth in scaffold	(Chen et al., 2021)
Microfl uidic technology	Microfluidic mixing	Small reaction solution volume and high production efficiency Controllable reaction and the size of the product can be controlled by adjusting the flow rate and pressure Simple reaction process Low cost Reduce tedious post purification processes	(Karnik et al., 2008)

#### 4.2.1 Injectable technology

**FIGURE 7:**
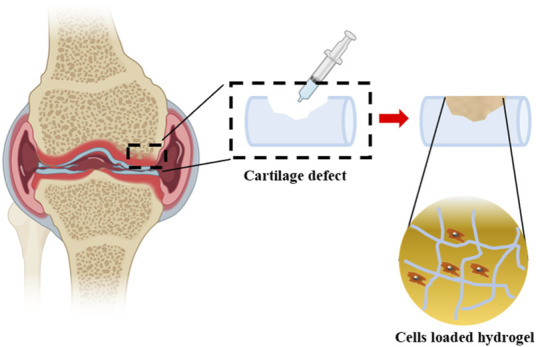
Cells loaded hydrogels are injected into cartilage defection. Partially designed with BioRender.

Given the irregular shape of the cartilage defect site, the injectable hydrogel has obtained great attention in the surgery, especially for minimally invasive surgery. The hydrogel scaffolds could be implanted into the cartilage defection by a simple and minimally invasive injection instead of the complex traditional treatment methods, such as microfractures, autologous chondrocyte transplantation, and allogeneic bone cartilage transplantation. The self-healing hydrogels can be best characterized when referring to their ability to return to their primary shape after any mechanical manipulations adapted shape and *in situ* cross-linking capability (Wang P. H. et al., 2022). It also acts as a scaffold for cellular infiltration and promotes the natural healing process (Figure 7).

A large number of polysaccharide materials, such as sodium alginate, chitosan and HA, are often used as ideal substances for injectable hydrogel by physical crosslinking and chemical crosslinking (Xiao et al., 2023; Zhu et al., 2017). The hydrogels combined with nanoparticles, exosomes and cells showed good reversibility under environmental stimuli and improved compliance of patients with cartilage injury (Zhang F. X. et al., 2021; Atoufi et al., 2019). Therefore, many hydrogels are responsive to environmental stimuli, that is, when the hydrogel is stimulated by light, temperature, and other environmental factors, its internal structure will undergo reversible changes, which will lead to changes in shape, permeability, and mechanical properties (Fallahi et al., 2022; Li X. et al., 2021; Weizel et al., 2020).

#### 4.2.2 3D bioprinting technology

**FIGURE 8:**
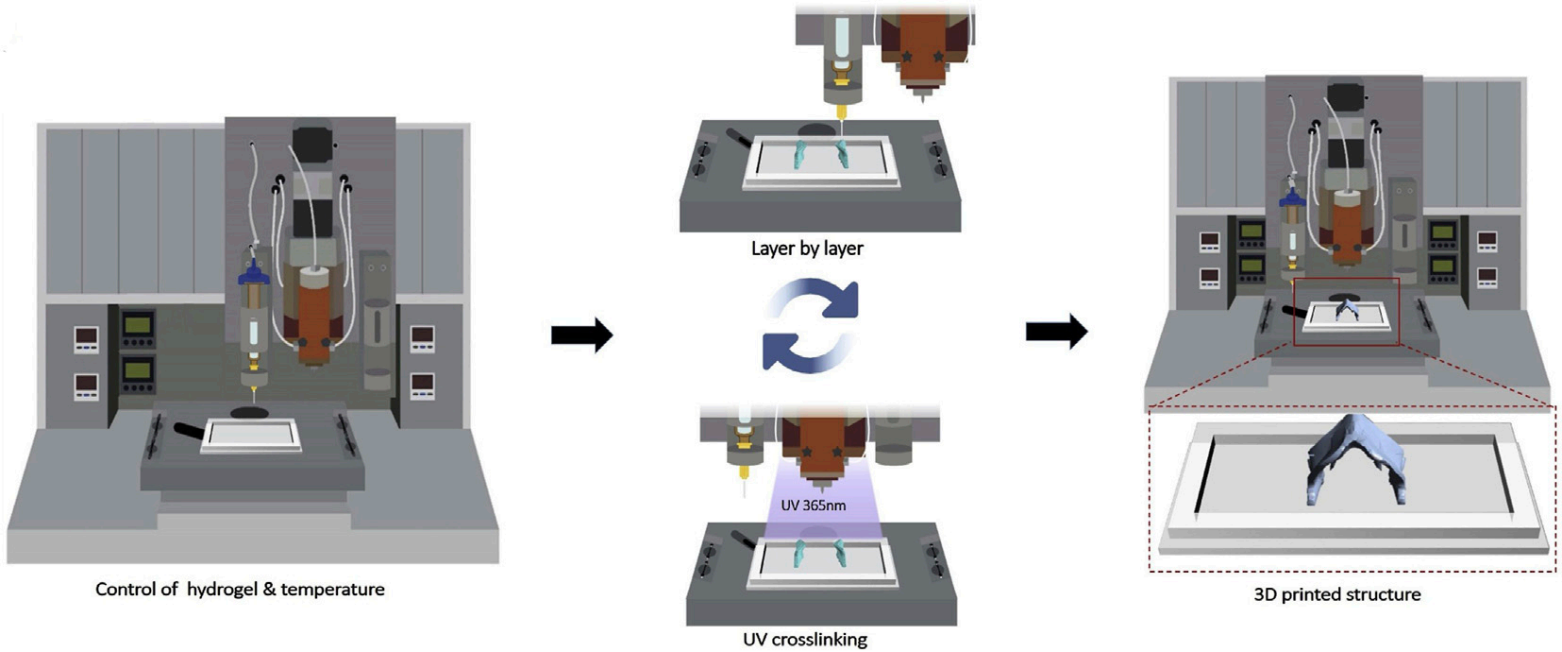
The scheme of 3D bioprinting process. The bioink solution was synthesized by GelMA, GMHA and cells, and then was printed on a 24-well culture plate with a nozzle of 0.25 mm (25 G) using a 3D Discovery Instrument. (Reprinted from Lee et al., 2020, with permission from Elsevier).

Compared with the uncontrollable injectable hydrogel directly injected into the cartilage defect site, 3D printing technology has unique performances in loading cells, exosomes and other active substances onto the hydrogel scaffold materials through layer-by-layer printing (Figure 8), so as to accurately treat the cartilage defect damage (Lee et al., 2020). It offers an exciting opportunity to fabricate biological constructs with specific geometries, clinically relevant sizes and functions for biomedical application. A limited number of carbohydrate polymer materials, including alginate, chitosan, and chondroitin sulfate could be designed as bio-ink for 3D scaffolds given the mechanical stability, viscoelastic behavior, printability, shape fidelity and structural integrity (Kim et al., 2018; Mihajlovic et al., 2022; Yang X. C. et al., 2018). The viscoelastic properties of sodium alginate and dECM alone could not meet the need for printability. TOCN was introduced into the combination to remarkably improve the viscosity and dECM/TOCN/alginate hydrogel scaffold based on SA by 3D printing technology (Zhe et al., 2023). On the one hand, the two-step crosslinking process could effectively improve the printability and mechanical strength. On the other hand, dECM and TOCN could provide the nutrient supply for cell proliferation and promote chondrogenesis.

#### 4.2.3 Microfluidic technology

**FIGURE 9:**
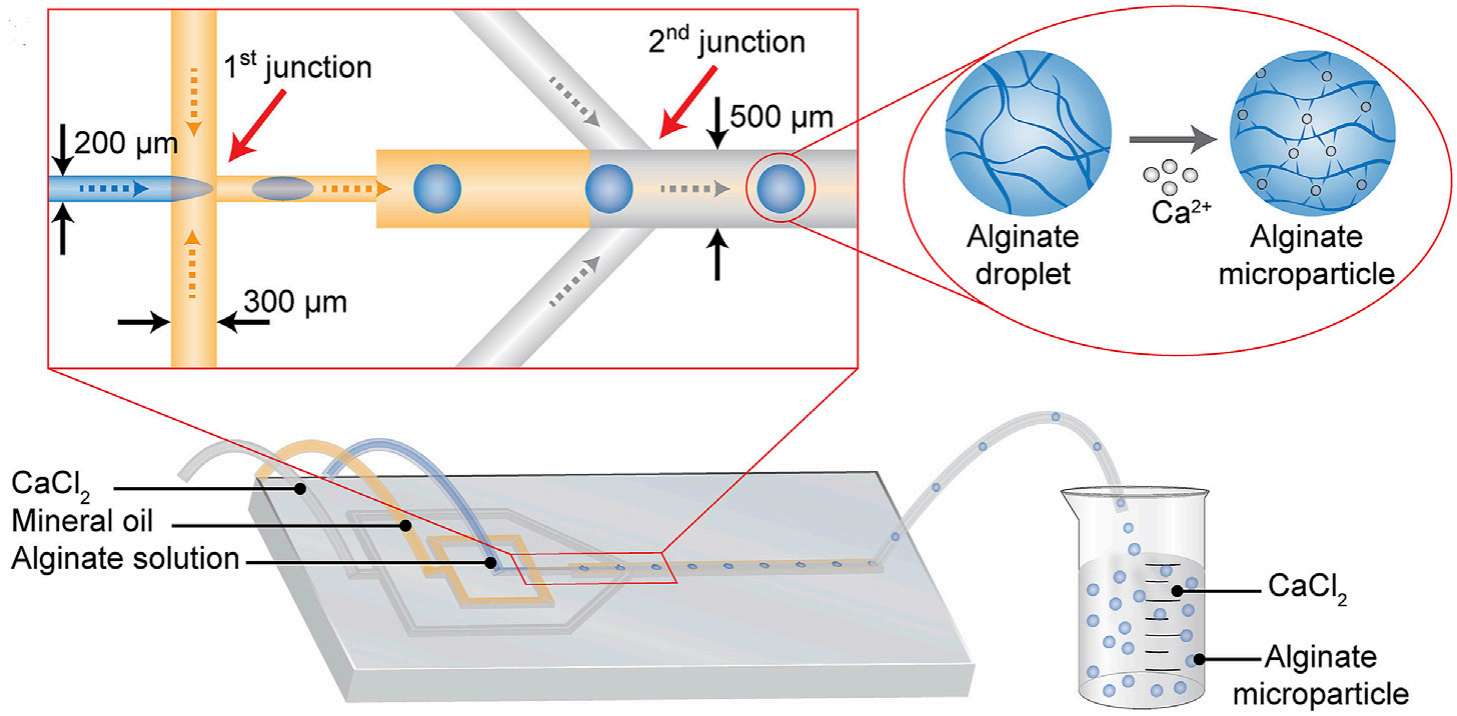
Schematic of synthesizing alginate microparticles in the microdevice. The mineral oil solution divides the sodium alginate solution into microsphere droplets at the 1st junction. And then the microsphere droplets are mixed with the calcium chloride solution at the 2nd junction, where CaCl_2_ solution crosslinks with sodium alginate to form sodium alginate microparticle. (Reprinted from Trinh et al., 2021, with permission from Elsevier).

Microfluidic is a kind of technology that could operate the fluid in the micrometer-sized chips to complete the processes, like material synthesis and reagent analysis. At present, there are many researches on the synthesis of cartilage regeneration using microfluidic devices (Saygili et al., 2021; Yao et al., 2023). Alginate particles with a controlled size range of 178–375 μm were synthesized based on the droplet microfluidic technique (Figure 9). In detail, the alginate droplet size was freely controlled in a microfluidic chip by changing the fluid conditions of alginate and oil, and then the alginate droplet gelated in the presence of calcium ions. Alginate based hydrogel loaded with transforming growth factor β3 (TGF-β3) can effectively induce chondrogenesis of human mesenchymal stem cells (Trinh et al., 2021).

### 4.3 The adhesion of hydrogels for cartilage regeneration

The good and stable integration between hydrogel implants and host tissue is crucial for cartilage scaffolds. Due to the weak adhesion of hydrogels to wet tissues, designing and manufacturing hydrogels with high adhesive strength remains a significant challenge in existing hydrogel technology for enhancing the regenerative capacity of cartilage defects. The physical and biochemical structures of natural polysaccharides almost fail to meet the high adhesion requirements of tissue engineering scaffolds. Utilizing their inherent properties, many innovative methods, including chemical, physical, or combined modifications, have been employed to improve their performance. For example, alginate sulfate-tyramine (ASTA) hydrogels with dual modifications of sulfation and tyraminization exhibit strong adhesion to cartilage tissue and have higher bonding strength compared to alginate-tyramine (AlgTA). Moreover, the expression of chondrogenic markers such as aggrecan and Sox9 genes in ASTA hydrogels is significantly higher than in AlgTA (Wang et al., 2014).

## 5 Conclusions and perspectives

Cartilage regeneration is a complex physiological process. The regeneration of cartilage tissue is not only dependent on the use of drugs, but also its biomechanical changes, cell adhesion growth, induction of chondrocyte differentiation, and regulation of the ECM microenvironment. In this review, polysaccharide-based hydrogels with different structures showing the various properties and functions in the process of cartilage regeneration were elaborated in detail. The discrepancies in molecules binding to polysaccharide-based hydrogels and the properties of hydrogels are responsible for the different efficiency in promoting the regeneration process of cartilage. It provides help and enlightenment for the preparation and application of polysaccharide-based hydrogels. Precisely mimicking the complex structure and composition of natural cartilage, with a greater focus on the interactions between cells, to promote better regeneration of cartilage tissue, is a consideration and development direction for future cartilage regeneration and repair. In addition to the mechanical performance, cell adhesion performance, and biocompatibility, efforts to improve the wet adhesion of hydrogels may also be considered for future cartilage defect repair.

Developing advanced technologies to synthesize polysaccharide-based hydrogels is essential and another aspect of future research. Considering the irregular shape of the cartilage defect site, injectable hydrogels are ideal candidates. And 3D printing technology and microfluidic technology have received increasing attention in the field. However, the hydrogel scaffold could not respond to stimuli similar to the body with the changes in the external environment, which is still the limitation of current research. 4D printing technology might be a direction for future development. It can not only customize the shape of materials on a personalized basis but also respond according to the characteristics of the physiological environment.

Besides, it is a huge challenge to clinical from laboratory. Therefore, it is necessary to further improve the preclinical trials to understand the metabolic and inflammatory processes of polysaccharide-based hydrogels *in vivo* as much as possible and to better understand the effects of carbohydrate hydrogels on cartilage regeneration processes.
